# Abundance indices and biological traits of juvenile salmon (*Salmo salar*) sampled in three rivers on the Atlantic and Channel coasts (France)

**DOI:** 10.3897/BDJ.5.e15125

**Published:** 2017-11-09

**Authors:** Frédéric Marchand, Laurent Beaulaton, Etienne Prévost, Richard Delanoë, Jean-Pierre Destouches, François Gueraud, Yoann Guilloux, Nicolas Jeannot, Emmanuel Huchet, Frédéric Lange, Jacques Rives, Julien Tremblay, Nadine Herrard, Didier Azam

**Affiliations:** 1 U3E, Ecologie et Ecotoxicologie aquatique,INRA, pôle Gest'Aqua, 35042 Rennes, France; 2 AFB, pôle Gest’Aqua, 35042 Rennes, France; 3 ECOBIOP, INRA, University Pau & Pays Adour, Aquapôle, Quartier Ibarron, 64310 Saint-Pée-sur-Nivelle, France; 4 Fédération du Morbihan pour la Pêche et la Protection du Milieu Aquatique, 56890 Saint-Avé, France

**Keywords:** abundance, biological traits, juvenile, coastal river, *Salmo salar*, salmon

## Abstract

**Background:**

Atlantic Salmon (*Salmo salar*) is an anadromous migratory species adapted to cool temperatures. It is protected by the Bern convention and by the European Habitats Directive. It has been listed as vulnerable by the French IUCN Red List. Salmon decline is the result of combined and cumulated, mainly anthropic, causes: climate change, increasingly high number of impoundments, degradation of water quality and habitat and over-exploitation by fisheries. Monitoring of this species has been carried out on three rivers in France (Southern part of the distribution area) to produce data and knowledge (growth, precocious maturity, survival) for stock management.

For 24 years, a specific and standardised electric fishing protocol has been used to target young-of-the-year (0+ parr) Atlantic salmon. Sampling was restricted to areas with shallow running water that flows over a coarse bottom substrate, i.e. the preferred habitat of young salmon. This monitoring and inventory of growing areas thus allows assessment of juvenile recruitment and provides baseline data required to calculate total allowable catches (TACs).

**New information:**

The dataset currently consists of 47,077 occurrence data points from 105 sites spanning up to 24 years in three different watersheds in France. Beyond our project, this dataset has a clear utility to research since it associates abundance measurements with the measurement of biological traits and the collection of tissue samples. It allows for current and retrospective characterisation of individuals or populations, according to life history traits and genetic features in relation to changes in environmental conditions. The fact that the monitoring takes place in France, the southern part of the distribution area, over 24 years, makes the dataset particularly relevant for climate change studies.

## Introduction

The Environmental Research Observatory (ERO) on Diadromous Fish in Coastal rivers (DiaPFC) is a research infrastructure focused on studying the evolution of diadromous fish populations under the influence of human-induced and environmental changes (mainly agriculture and climate). Currently, coastal rivers are the main refuge for diadromous fish that have disappeared or dramatically decreased in larger rivers (30,000 fish caught on the Loire-Allier system in the 1890s (Bachelier R. 1963) to less than 1500 counted (source: http://www.logrami.fr/actions/stations-comptage). A survey is conducted each year in early autumn (late September to early October) to quantify the abundance of juvenile Atlantic salmon in three rivers on the Atlantic and Channel coasts: the Oir in Normandy, the Scorff in Brittany and the Nivelle in the Basque Country. The survey began in 1993 in the Oir and the Scorff and in 2003 in the Nivelle. A specific and standardised electric fishing protocol, Salmon Abundance Index (Prévost and Baglinière 1995) is being used to target young-of-the-year (0+ parr) Atlantic salmon, but older fish (juveniles ≥1+) are also caught and included in the dataset. The same protocol is being applied throughout the time series. It allows the dataset to be used for studies at the watershed scale (connectivity, growth area), stock evolution across time or to compare rivers from different regions to evaluate local versus global changes. The Oir, Scorff and Nivelle datasets contain a total of 1755 sampling events. The data consists of abundance indices and biological traits measured for the fish sampled: sex, maturity status, length, weight and age. The data have been used to develop predictive models (Rivot and Prévost 2002, Piou and Prévost 2013, Buoro et al. 2012) and tools to provide scientific advice to improve management of this heritage species (Bal 2011, Buoro et al. 2010, Gregory et al. 2017). Samples have also been used for study of the long-term effect of nitrogen loads on carbon cycling in rivers with stable isotope analyses on archived fish samples (Roussel et al. 2014).

## Project description

### Title

Environmental Research Observatory (ERO) on Diadromous Fish in Coastal rivers (DiaPFC): Abundance indices and biological traits of juvenile salmon (*Salmo salar*).

### Study area description

**Figure 1.**
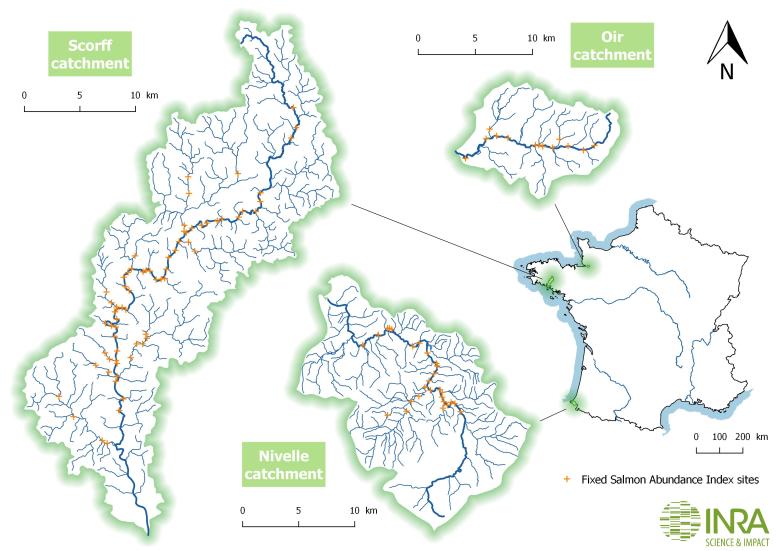
Geographic location of the three coastal rivers in which the dataset was collected.

The Environmental Research Observatory (ERO) on Diadromous Fish in Coastal rivers (DiaPFC) monitors three coastal rivers on the Atlantic and Channel coasts of France: the Oir in Normandy, the Scorff in Brittany and the Nivelle in the French Basque Country (Fig. 1).

Oir river: Latitude ranges from 48.6840 to 48.5985; longitude ranges from -1.2949 to -1.0994 and elevation range from 9 and 80 metres.

The Oir River is located on the southern edge of Normandy. It is a tributary of the Sélune, a coastal river that flows into the Bay of Mont Saint-Michel. The Oir is 21km long and has a mean gradient of 1.1%. Annual mean discharge is 1.1m^3^/s and the drainage basin area is 85.4km^2^. Geologically, the basin is dominated by sedimentary schist and metamorphic hornfels with granite inclusions. The water has nearly neutral pH and reasonably good quality except for high nitrate concentrations. Agriculture is the main human activity and land use. Migratory fish cannot access the upper Sélune because a dam 15km from the sea blocks their movement. The Oir, with 12km accessible to Atlantic salmon, is the main spawning ground and most productive tributary of the Sélune hydrographic network.

Scorff River: Latitude ranges from 47.7718 to 48.1682; longitude ranges from -3.2497 to -3.3404 and elevation range from 0 and 144 metres.

The Scorff River is a small coastal river in southern Brittany (France). The main river is 78.6km long, including a 15km estuary. The mean gradient is 3.6%, annual mean discharge is 5m^3^/s and the drainage basin area has an area of 480km^2^. Agriculture is the main human activity and land use, with several areas of moors and forests. Atlantic salmon essentially colonise only a 50km stretch of the main river starting at the head of the estuary. Most reproduction of Atlantic salmon occurs in the main river (Bagliniere 1979).The Scorff has only three larger tributaries. Until recently, Atlantic salmon could colonise only downstream sections of these tributaries due to mill dams.

Nivelle River: Latitude ranges from 43.2426 to 43.3912; longitude ranges from -1.4799 to -1.6694 and elevation range from 6 and 75 metres.

The Nivelle River is a 39km long coastal river in the Basque Country. Its source lies in Spain and it flows into the Bay of Biscay at Saint-Jean-de-Luz. Its drainage basin has an area of 238km^2^, which is dominated by marly-calcareous formations. It is essentially agro-pastoral with more than 50% of the land area consisting of moors (Dumas 2003). The oceanic climate, mild and wet (rainfall of 1700mm/yr in St-Pée-sur-Nivelle), provides a mean annual discharge of 5.4m^3^/s downstream of the confluence of the main tributary, the Lurgorrieta and 9m^3^/s at the mouth. The water, neutral to slightly alkaline, is of good quality upstream of Saint-Pée-sur-Nivelle, but degrades downstream.

### Design description

Rivers are equipped with diadromous fish trapping facilities and have been thoroughly and continually surveyed since the mid-1980s. They are affiliated with experimental ecology facilities located in Rennes (Brittany) and Saint-Pée-sur-Nivelle (Basque Country). To quantify their abundance of juvenile Atlantic salmon, they are surveyed every year in early autumn (late September to early October). The electric fishing protocol of Prévost and Baglinière (1995) is used to monitor the fish. It targets young-of-the-year (0+ parr) Atlantic salmon, but older fish (juveniles ≥1+) are also caught and included in the dataset. This protocol is not a Water Framework Directive standard protocol but has been calibrated with density which allows comparisons with other protocols. Sampling is restricted to areas with shallow running water that flows over a coarse bottom substrate, i.e. the preferred habitat of 0+ parr. The data consist of abundance indices and biological traits measured for the fish sampled: sex, maturity status, length, weight and age. Scale samples are taken from all fish for which size is not well correlated with age (Baglinière et al. 1985;Baglinière and Le Louarn 1987).

## Sampling methods

### Study extent

In the Oir, sampling is conducted at 11 sites along the mainstream and 2 others on the 2 major tributaries (Fig. 1). In the Scorff, sampling is conducted at 42 sites along the mainstem and 24 sites distributed over 12 tributaries (Fig. 1). In the Nivelle, sampling is conducted at 20 sites along the mainstem, 6 sites along the main tributary (i.e. the Lurgorrieta) and 2 other sites distributed over 2 smaller tributaries (Fig. 1). All sites are located in the area of the hydrographic network colonised by Atlantic salmon and visited once a year at the beginning of autumn (end of September to early October). Sampling by site and by year is indicated in Suppl. material 1.

### Sampling description

Salmon Abundance Index is described in Prévost and Baglinière (1995). Since the beginning of data collection, fish have been caught with the same backpack electrofishing equipment (Martin Pêcheur®, DREAM Electronique, Pessac, France) tuned to produce a pulsed DC with 400 Hz frequency, 250-300 volts and a square-waveform 4-10% duty cycle. Fishing consists of the following steps:

1. Two large dip-nets with semi circular metal frames, one 60cm wide and 40cm high and another 80cm wide and 40cm high with 4mm² mesh are placed facing the current, are rested on the bottom and are maintained in a fixed position. Dip-nets never varied over time.
2. The area shocked is 4-5m directly upstream of the stationary dip nets so that fish disabled by the shocker are carried by the current into the dip nets.
3. Fish attracted to the anode and shocked go down into the nets guided by the electrode and driven by the water flow. When necessary, a small hand net is used to catch fish stuck on the bottom or in aquatic vegetation.
4. Individuals are transferred into a bucket previously filled with water.
5. The entire team moves laterally several metres from the area that was recently disturbed by the electric field; the carrier of the electrofishing equipment is careful not to step into the area the anode will next explore . When a bank is reached, the team moves a few metres upstream.

Steps 1-5 are repeated. Sampling at a given site stops after 5 minutes of fishing, i.e. the duration during which the electric field is applied in the water, this being measured directly on the counter of the electrofishing equipment. Juvenile abundance is quantified by the number of individuals captured per unit effort (5 minutes of fishing under the conditions specified above).

Biometric measurements of the fish are recorded after anaesthetising specimens with a solution of benzocaïne (Neiffer and Stamper 2009, Gilderhus 1989). Then length is measured from the tip of the mouth to the fork of the caudal fin (1mm precision). Fish are then weighed (0.2g precision), sex and certainty of maturity status is assessed by applying gentle pressure to the belly, which expresses sperm from mature males (the individual can be mature without sperm detection). After recovery from anaesthesia, all fish sampled are then released at their site of capture.

Age is estimated by scale analysis according to standard methods described in *Richard and Bagliniere (1990), Baglinière and Le Louarn (1987)Baglinière et al. (1985)*.

### Quality control

Data are stored in a PostgreSQL database and are thus subject to an integrity check by the database management system. Consistency checks, mainly on size and weight, were performed when the field records were entered. Since 2016, length and weight are automatically measured and sent by bluethooth to avoid transcription errors. All our weighing devices are checked annually and the tool length measurement is calibrated at the beginning of a fishing session.

Use of the French National Service for Water Data and Reference Dataset Management (SANDRE, Service d'administration nationale des données et référentiels sur l'eau) guarantees interoperability with French water information systems. The Sandre is organised in a network of SIE-contributing institutes that bring their thematic knowledge, participate in the reference data sets management and ensure the overall coherence. Managed by AFB, this network is supported by a technical secretariat provided by the International Office for Water, which moves, develops and makes available these reference data sets.

For publication on the GBIF portal, data and metadata were transformed to compliance with Darwin Core standards Wieczorek et al. 2012 (Event Core and Occurrence Extension). These Global Biodiversity Information Facility datasets are Darwin Core Archive files, encoded in UTF-8 and permanently available on the GBIF portal.

## Geographic coverage

### Description

Observations included in this dataset originate from three coastal rivers on the Atlantic and Channel coasts of France: the Oir in Normandy, the Scorff in Brittany and the Nivelle in the Basque Country (Fig. 1).

### Coordinates

43.2426 and 48.6840 Latitude; -1.0994 and -3.3404 Longitude.

## Taxonomic coverage

### Description

Fundamental features of the specie's life cycle, distribution and ecology can be found in *Les poissons d’eau douce de France* (Keith et al. 2011).

### Taxa included

**Table:** 

Rank	Scientific Name	
kingdom	Animalia	
phylum	Chordata	
class	Actinopterygii	
order	Salmoniformes	
family	Salmonidae	
species	*Salmo salar* (Linnaeus, 1758)	Atlantic salmon

## Temporal coverage

### Notes

Field sampling and data collection have been conducted annually in September or October since 1993 for the Oir and Scorff and since 2003 for the Nivelle (Suppl. material 1).

## Collection data

### Collection name

Banque de données d'échantillons ichtyologiques

### Collection identifier

BDEI

### Specimen preservation method

Scales in envelopes and other tissues in alcohol

### Curatorial unit

3234 samples of scales 17969 samples of tissue

## Usage rights

### Use license

Creative Commons Public Domain Waiver (CC-Zero)

## Data resources

### Data package title

Abundance indices and biological traits of juvenile salmon (Salmo salar) sampled in three rivers on the Atlantic and Channel coasts (France)

### Number of data sets

3

### Data set 1.

#### Data set name

Abundances and biological traits of the juveniles salmon sampled in the survey of Salmon abundance Indices in the Oir river (France).

#### Data format

Darwin Core Archive format

#### Number of columns

36

#### Download URL

https://doi.org/10.15468/cjsjrj

#### Description

The dataset consists of two types of data:

- Sampling events, describing the protocol, date and capture location, which can be common to several captures. Events with no catch are also recorded and Occurrences associated with these events.
- Measurement or fact describing characteristics of each individual.

**Data set 1.** 

Column label	Column description
ID	Identifier of the occurence (GBIF)
institutionCode	The acronym in use by the institution having custody of data
collectionCode	The name identifying the data set from which the record was derived
ownerInstitutionCode	Institution having ownership of the data
basisOfRecord	The specific nature of the data record
occurrenceID	Identifier of the occurrence (INRA)
organismQuantity	Number of organism for the occurenceID
organismQuantityType	Type of quantification system used for the quantity of organisms
sex	Sex of the individual corresponding to the occurrence when it is possible (mainly male if it had a soft and distended underbelly from enlarged gonads or produced milt when massaged gently)
establishmentMeans	The process by which the biological individual(s) represented in the Occurrence became established at the location ("native", "cultivated",...)
occurrenceStatus	A statement about the presence or absence of a Taxon at a Location
eventID	Identifier of the event
samplingProtocol	Name of the protocol used during the event Name of the protocol used during the event
sampleSizeValue	Numeric value for the samplingEffort
sampleSizeUnit	The unit of measurement of the samplingEffort
samplingEffort	The amount of effort expended during an Event
eventDate	Date the event was recorded (aaaa-mm-dd)
locationID	Identifier for the location in which the event occurred
waterBody	Name of the water body in which the event occurred
country	Name of the country in which the event occurred
countryCode	Code of the country in which the event occurred
minimumElevationInMeters	Minimum elevation in metres
maximumElevationInMeters	Maximum elevation in metres
decimalLatitude	Geographic latitude
decimalLongitude	Geographic longitude
geodeticDatum	Spatial reference system
scientificName	Full scientific name of the species corresponding to the occurrence
kingdom	The full scientific name of the kingdom in which the taxon is classified
phylum	The full scientific name of the phylum in which the taxon is classified
class	The full scientific name of the class in which the taxon is classified
order	The full scientific name of the order in which the taxon is classified
family	The full scientific name of the family in which the taxon is classified
taxonRank	The taxonomic rank of the most specific name in the scientificName
measurementType	The nature of the measurement (length, weight, age, maturity, scales sampled, fin tissue sampled and number of associated occurences in the locationID)
measurementValue	The value of the measurement
measurementUnit	The units associated with the measurementValue

### Data set 2.

#### Data set name

Abundances and biological traits of the juveniles salmon sampled in the survey of Salmon abundance Indices in the Scorff river (France).

#### Data format

Darwin Core Archive format

#### Number of columns

36

#### Download URL

https://doi.org/10.15468/mz4lyw

#### Description

The dataset consists of two types of data:

- Sampling events, describing the protocol, date and capture location, which can be common to several captures. Events with no catch are also recorded and Occurrences associated with these events.
- Measurement or fact describing characteristics of each individual.

**Data set 2.** 

Column label	Column description
ID	Identifier of the occurence (GBIF)
institutionCode	The acronym in use by the institution having custody of data
collectionCode	The name identifying the data set from which the record was derived
ownerInstitutionCode	Institution having ownership of the data
basisOfRecord	The specific nature of the data record
occurrenceID	Identifier of the occurrence (INRA)
organismQuantity	Number of organism for the occurenceID
organismQuantityType	Type of quantification system used for the quantity of organisms
sex	Sex of the individual corresponding to the occurrence when it is possible (mainly male if it had a soft and distended underbelly from enlarged gonads or produced milt when massaged gently)
establishmentMeans	The process by which the biological individual(s) represented in the Occurrence became established at the location ("native", "cultivated",...)
occurrenceStatus	A statement about the presence or absence of a Taxon at a Location
eventID	Identifier of the event
samplingProtocol	Name of the protocol used during the event Name of the protocol used during the event
sampleSizeValue	Numeric value for the samplingEffort
sampleSizeUnit	The unit of measurement of the samplingEffort
samplingEffort	The amount of effort expended during an Event
eventDate	Date the event was recorded (aaaa-mm-dd)
locationID	Identifier for the location in which the event occurred
waterBody	Name of the water body in which the event occurred
country	Name of the country in which the event occurred
countryCode	Code of the country in which the event occurred
minimumElevationInMeters	Minimum elevation in metres
maximumElevationInMeters	Maximum elevation in metres
decimalLatitude	Geographic latitude
decimalLongitude	Geographic longitude
geodeticDatum	Spatial reference system
scientificName	The full scientific name of the class in which the taxon is classified
kingdom	The full scientific name of the kingdom in which the taxon is classified
phylum	The full scientific name of the phylum in which the taxon is classified
class	The full scientific name of the class in which the taxon is classified
order	The full scientific name of the order in which the taxon is classified
family	The full scientific name of the family in which the taxon is classified
taxonRank	The taxonomic rank of the most specific name in the scientificName
measurementType	The nature of the measurement (length, weight, age, maturity, scales sampled, fin tissue sampled and number of associated occurences in the locationID)
measurementValue	The value of the measurement
measurementUnit	The units associated with the measurementValue

### Data set 3.

#### Data set name

Abundances and biological traits of the juveniles salmon sampled in the survey of Salmon abundance Indices in the Nivelle river (France).

#### Data format

Darwin Core Archive format

#### Number of columns

36

#### Download URL

https://doi.org/10.15468/alsjvy

#### Description

The dataset consists of two types of data:

- Sampling events, describing the protocol, date and capture location, which can be common to several captures. Events with no catch are also recorded and Occurrences associated with these events.
- Measurement or fact describing characteristics of each individual.

**Data set 3.** 

Column label	Column description
ID	Identifier of the occurence (GBIF)
institutionCode	The acronym in use by the institution having custody of data
collectionCode	The name identifying the data set from which the record was derived
ownerInstitutionCode	Institution having ownership of the data
basisOfRecord	The specific nature of the data record
occurrenceID	Identifier of the occurrence (INRA)
organismQuantity	Number of organism for the occurenceID
organismQuantityType	Type of quantification system used for the quantity of organisms
sex	Sex of the individual corresponding to the occurrence when it is possible (mainly male if it had a soft and distended underbelly from enlarged gonads or produced milt when massaged gently)
establishmentMeans	The process by which the biological individual(s) represented in the Occurrence became established at the location ("native", "cultivated",...)
occurrenceStatus	A statement about the presence or absence of a Taxon at a Location
eventID	Identifier of the event
samplingProtocol	Name of the protocol used during the event Name of the protocol used during the event
sampleSizeValue	Numeric value for the samplingEffort
sampleSizeUnit	The unit of measurement of the samplingEffort
samplingEffort	The amount of effort expended during an Event
eventDate	Date the event was recorded (aaaa-mm-dd)
locationID	Identifier for the location in which the event occurred
waterBody	Name of the water body in which the event occurred
country	Name of the country in which the event occurred
countryCode	Code of the country in which the event occurred
minimumElevationInMeters	Minimum elevation in metres
maximumElevationInMeters	Maximum elevation in metres
decimalLatitude	Geographic latitude
decimalLongitude	Geographic longitude
geodeticDatum	Spatial reference system
Spatial reference system	Full scientific name of the species corresponding to the occurrence
kingdom	The full scientific name of the kingdom in which the taxon is classified
phylum	The full scientific name of the phylum in which the taxon is classified
class	The full scientific name of the class in which the taxon is classified
order	The full scientific name of the order in which the taxon is classified
family	The full scientific name of the family in which the taxon is classified
taxonRank	The taxonomic rank of the most specific name in the scientific Name
measurementType	The nature of the measurement (length, weight, age, maturity, scales sampled, fin tissue sampled and number of associated occurences in the locationID)
measurementValue	The value of the measurement
measurementUnit	The units associated with the measurementValue

## Additional information

These datasets are intended to be updated annually. A new DOI should be attributed to each update.

All monitoring activities of the ERO DiaPFC are submitted to and accepted by an animal ethics committee.

## Supplementary Material

Supplementary material 1 Sampling events by site and by year Data type: Event
File: oo_166224.xlsx Frédéric Marchand
